# Design and Fabrication of Polymeric Hydrogel Carrier for Nerve Repair

**DOI:** 10.3390/polym14081549

**Published:** 2022-04-11

**Authors:** Xiaoyu Ma, Mengjie Wang, Yuanyuan Ran, Yusi Wu, Jin Wang, Fuhai Gao, Zongjian Liu, Jianing Xi, Lin Ye, Zengguo Feng

**Affiliations:** 1 School of Materials Science and Engineering, Beijing Institute of Technology, Beijing 100081, China; 3120201170@bit.edu.cn (X.M.); sainfeng@bit.edu.cn (Z.F.); 2 School of Beijing Rehabilitation Medicine, Capital Medical University, Beijing 100044, China; meng_jie_w@163.com; 3 Department of Rehabilitation, Beijing Rehabilitation Hospital, Capital Medical School, Beijing 100044, China; a202143@ccmu.edu.cn (Y.R.); gfh234123@163.com (F.G.); 4 Key Laboratory of Multifunctional Nanomaterials and Smart Systems, Suzhou Institute of Nano-Tech and Nano-Bionics, Chinese Academy of Sciences, Suzhou 215123, China; na803655@student.reading.ac.uk (Y.W.); jwang2014@sinano.ac.cn (J.W.); 5 NUIST-UoR International Research Institute, Reading Academy, Nanjing University of Information Science and Technology, Nanjing 210044, China

**Keywords:** hydrogel, nerve repair, carrier, fabrication, stroke

## Abstract

Nerve regeneration and repair still remain a huge challenge for both central nervous and peripheral nervous system. Although some therapeutic substances, including neuroprotective agents, clinical drugs and stem cells, as well as various growth factors, are found to be effective to promote nerve repair, a carrier system that possesses a sustainable release behavior, in order to ensure high on-site concentration during the whole repair and regeneration process, and high bioavailability is still highly desirable. Hydrogel, as an ideal delivery system, has an excellent loading capacity and sustainable release behavior, as well as tunable physical and chemical properties to adapt to various biomedical scenarios; thus, it is thought to be a suitable carrier system for nerve repair. This paper reviews the structure and classification of hydrogels and summarizes the fabrication and processing methods that can prepare a suitable hydrogel carrier with specific physical and chemical properties. Furthermore, the modulation of the physical and chemical properties of hydrogels is also discussed in detail in order to obtain a better therapeutic effect to promote nerve repair. Finally, the future perspectives of hydrogel microsphere carriers for stroke rehabilitation are highlighted.

## 1. Introduction

**Figure 1:**
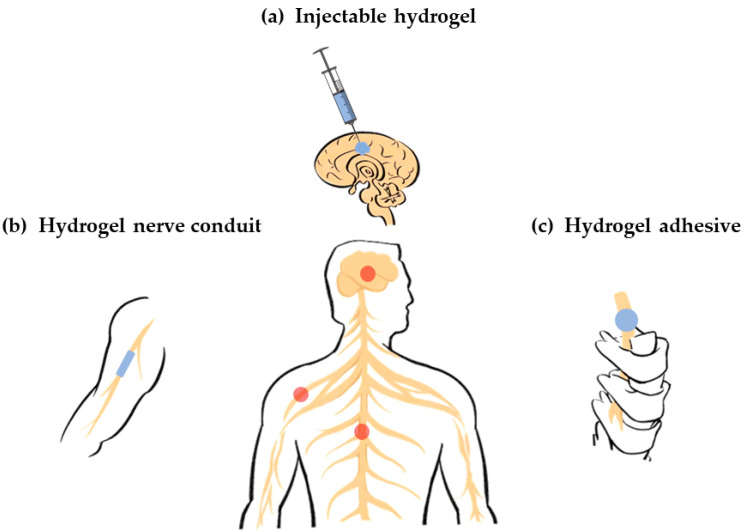
Various hydrogel carriers for nerve repair. (**a**) Injectable hydrogel carriers for brain nerve repair (central nervous system); (**b**) Hydrogel carriers as conduits for peripheral nerve repair (peripheral nervous system); (**c**) Hydrogel carriers as adhesive for spinal cord repair (central nervous system).

Nerve repair still remains a huge challenge in the clinic. Recently, more and more researchers have paid attention to the significant effects of biomaterials such as hydrogels on nerve repair. The nervous system is divided into the central nervous system (CNS), which is composed of the brain nerve and spinal cord, and peripheral nervous systems (PNS) [1,2]. As shown in Figure 1, hydrogels are widely used in various nervous systems, including the CNS and PNS, but are particularly useful in repairing brain nerves after stroke.

Recently, the incidence of stroke worldwide has sharply increased. It has a very poor prognosis, including a high mortality and long-term disability. Stroke patients often show difficulties with speaking, cognitive impairment, memory loss, dementia, and depression, which indicate the damage and loss of brain nerves [3,4,5]. Thus, it is believed that brain nerve repair after stroke plays a vital role in patient rehabilitation and body function reconstruction. Currently, stroke could be treated to some extent in the acute phase in the clinic [6]. However, the therapeutic time window is so narrow that the most patients miss it. Thus, rehabilitative therapies in the sub-acute phase and chronic phase that promote nerve repair and functional recovery are very important in stroke treatment. Although some approaches, including neuroprotective agents, stem cells and immune T-cells, as well as various growth factors, are shown to be effective in animal models, some concerns still remain [7,8,9]. As these therapeutic substances are usually intravenously injected, it is difficult to reach the brain site and maintain enough local concentration due to the presence of the blood –brain barrier in sub-acute phase and chronic phase and the lack of targeting ability [10]. Although therapeutic substances could also be delivered by cephalotomy or craniotomy to endow a high initial concentration, they would be absorbed quickly by the cerebrospinal fluid, leading to a sharp decrease in local concentration. Consequently, a biocompatible carrier system, which could effectively deliver therapeutic substances and maintain their long-term retention in the brain site, is highly desirable for stroke treatment in the sub-acute and chronic phases. 

Hydrogel is a water-swollen and cross-linked polymeric network produced by the simple reaction of one or more monomers [11]. They possess a degree of flexibility that ios very similar to natural tissues due to their large water content and exhibit a 3D structure that emulates the properties of the native extracellular matrix (ECM) within the CNS [12,13]. A hydrogel is considered to be an ideal carrier system for various biomedical applications including nerve repair. Extensive studies have revealed that hydrogel carriers exhibit an excellent restorative function against stroke-induced brain damage [14,15]. Thus, it is necessary to make a comprehensive summary to provide useful insights and technical enlightenment for researchers to optimize the design and fabrication of hydrogel carriers in order to achieve better therapeutic effects in nerve repair, which might be translated into the clinic in the future. Furthermore, hydrogels that can be injected in form of liquid and gelate in situ by some external stimuli, including heat, light, magnetism, etc., have great potential to be very suitable carriers for brain nerve repair after stroke. The liquid formed during the injection greatly simplifies both the loading and the delivering of various therapeutic substances. Then, the in situ gelation causes sustainable release behavior to maintain long-term retention in brain, resulting in a high bioavailability, as well as good therapeutic effect. On the other hand, the field of brain nerve repair after stroke urgently needs a breakthrough in both science and technology to drive it from the lab to the clinic due to the sharp increase in the number of stroke patients worldwide. Consequently, this review pays particular attention to summarizing and highlighting the design, synthesis and application of various hydrogel systems for stroke rehabilitation, while other hydrogel systems for spinal cord and PNS repair are also included in the scope of this review. First, the classification, loading capacity and fabrication of hydrogel systems for nerve repair are summarized in detail in this review. Then, ways to regulate the physical properties of the hydrogel system to better meet the requirements of nerve repair are discussed. 

## 2. Classification of Hydrogel Carriers for Nerve Repair

A hydrogel exhibits several advantages in its application as a nerve repair material. First of all, it is an ideal carrier that can load cells, drugs, nutrients, growth factors and other bioactive components, as well as maintain its activity for a long time to promote nerve repair. Second, it has good biodegradability so that it can provide support for the growth of new tissues at the initial stage of repair and provide space for nerve regeneration through gradual degradation. Third, it has a higher moisture content and better biocompatibility, making it a suitable substrate for endogenous or transplanted cell growth. Forth, it has an adjustable mechanical strength, which can flexibly fit the modulus of the damaged nerve tissue. Finally, it has abundant pore space that is conducive to cell growth and material exchange. Therefore, the application of hydrogels in the field of nerve repair has been widely studied. 

From different angles, hydrogels have various classification standards. For instance, based on their physical shape, hydrogels can generally be classified as blocks, microspheres, fibrous, and films. Based on their physical structure, hydrogels can be divided into three categories: amorphous, crystalline and semicrystalline phases, which is a mixture of amorphous and crystalline. Based on the presence or absence of electrical charge located on the cross-linked chains, hydrogels can be divided into four categories: nonionic, ionic, amphoteric electrolyte and zwitterionic. Based on the cross-linking mechanism, hydrogels can be divided into two categories. The chemical cross-linked hydrogel network is a permanent connection. The physical cross-linked hydrogel network is a transient connection that arises from molecular/polymer chain entanglement or under the action of weak interactions, such as hydrogen bonding and electrostatic interactions. Based on their polymeric composition, hydrogels can be divided into three categories. Homopolymeric hydrogels are referred to as polymer networks derived from a single species of monomer. Copolymeric hydrogels are referred to as polymer networks that comprise two or more different monomer species with at least one hydrophilic component. Multipolymer interpenetrating polymeric hydrogels are referred to as polymer networks made of two independent cross-linked components where one component is a cross-linked polymer and other component is a non-cross-linked polymer [11]. 

**Figure 2:**
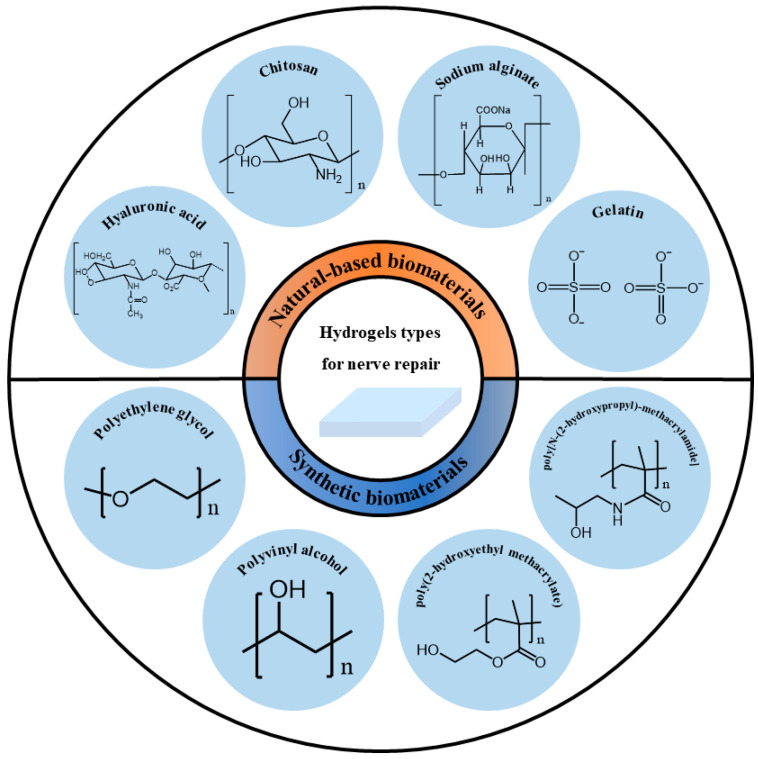
Classification of hydrogel carriers for nerve repair based on their sources.

Based on their source, hydrogels can be divided into natural-based biomaterials and synthetic biomaterials. The source of the polymer has a greater impact on the biocompatibility and safety of the hydrogel when considered as a functional material for biological tissues. Therefore, this article introduces the classification of hydrogels based on their sources as shown in Figure 2.

### 2.1. Natural-Based Biomaterials

Natural-based biomaterials refer to biomaterials generated under natural conditions, usually in the form of naturally occurring macromolecular substances derived from animals, plants or humans. Natural-based biomaterials can be gradually degraded into small molecular substances existing in the body through hydrolysis, enzymatic hydrolysis and other methods under the physiological environment of the body, and then completely absorbed or excreted through metabolism without causing toxic side effects on the body itself. Because of their large availability, large quantities of natural-based biomaterials are accessible at reasonable prices. Thus, they have been widely used as hydrogels for nerve repair. However, their mechanical properties are usually poor, and some of them are insoluble in water or common organic solvents. It is difficult to purify them for large-scale production [16]. The following will briefly introduce and discuss some natural-based biomaterials used in nerve repair.

Hyaluronic acid (HA) is a macromolecular linear polysaccharide and the main component of an extracellular matrix, especially abundant in the central system. It is involved in the regulation of a variety of cell activities. Its unique molecular structure and physicochemical properties endows it with a variety of important physiological functions in the body [17]. Thus, it has been widely used for nerve repair. In vitro experiments have shown that HA hydrogel can promote strong neurite outgrowth [18]. Neurite is an elongated part extending from the cell body of a nerve cell (neuron), and can be divided into dendrites and axons. The dendrite is the entrance that receives information, and the axon is the exit that sends out information. Both are used together for information transfer between cells. HA hydrogels also support the viability of neural precursor cells [19,20], neural stem cells [21] and Schwann cells [22], maintain their activities, and enable them to proliferate and differentiate. HA hydrogels have a high water-absorbency and enzyme degradability. In vivo, they usually exhibit rapid erosion and degradation behavior. They mainly form a hydration open mesh at the site of injury to inhibit the formation of a glial scar. Glial scarring is a compensatory reaction product after injury. In the early post-injury period, glial scarring can limit the spread of inflammation. However, with the progression of the disease, a glial scar can form a physical and chemical barrier, secrete a variety of axon regeneration inhibitory factors, and hinder the recovery of neural function. By reducing its formation, glial scarring can promote neuroprotection and functional recovery. In addition, HA hydrogels support the cell infiltration of cells into the hydrogel and angiogenesis [23]. Broguiere cross-linked high-molecular-weight hyaluronic acid and transglutaminase to prepare a new type of hydrogel, which could be covalently bound to the protein in the body when it was injected in vivo, so that the hydrogel could covalently bind to brain or spinal cord defects. Thus, it showed a fast neurite outgrowth, axonal and dendritic speciation, strong synaptic connectivity in 3D networks, and rapidly occurring and long-lasting coordinated electrical activity [24]. The healing potential of HA hydrogels may be partly attributed to hyaluronic acid promoting interactions between hydrogels and the central nervous system [25].

Collagen is the most important extracellular water-insoluble fibrin. It is the skeletal component of the extracellular matrix and the most abundant protein in the human body. Collagen hydrogel can be used as the inner filling of nerve conduits to repair broken nerve tissue [26] or independent tissue engineering material to fill tissue defect [27]. It is an excellent carrier of neural stem cells, mesenchymal stem cells and other cells. It can be used for cell transplantation to repair brain or spinal cord injury [28,29]. It can also be used as a sustained-release drug carrier [30]. 

Gelatin is obtained by the thermal denaturation of collagen. Gelatin has a relatively low antigenicity compared with collagen. The body’s immune system response can be avoided or reduced during its use. Animal-derived gelatin has been widely studied in medical applications due to its biocompatibility, plasticity and adhesion. Gelatin hydrogel is beneficial for cell adhesion and activity, and has significant effects on nerve healing and reconstruction [31]. Experimental results have shown that the nerve conduit containing gelatin hydrogel and a basic fibroblast growth factor promoted axonal regeneration after a 15 mm sciatic nerve injury in rats. The nerve regeneration rate was significantly increased, and a large number of regenerated nerve axons were induced [32]. However, this effect was not as good as autologous transplantation [33]. Due to its weak mechanical strength and rapid degradation, most gelatins will be prepared in a combination with other biomaterials to improve the comprehensive properties of hydrogels [34,35,36]. 

Fibrin is a protein formed during blood coagulation and is also a component of the extracellular matrix. Fibrin has an obvious advantage over other types of protein hydrogels in the repair of the nervous system: when autologous fibrin is used, it has a complete biocompatibility [37]. Fibrin hydrogel can improve the survival rate of neural stem cells [38,39], support the neurogenic differentiation of mesenchymal stem cells and the regeneration of axons [40]. It also has a satisfactory effect in loading drugs [41]. In order to overcome the application limitation of fibrin, Robinson crosslinked it with genipin to reduce its degradation rate, so that the hydrogel scaffold could support the neural aggregates derived from neural progenitor cells and induce neurite outgrowth [42].

Keratin is a kind of fibrin with abundant sources and biological activities [43]. Keratin hydrogel not only has a good cell adhesion [44] but can also maintain the high activity of cells [45,46]. Keratin hydrogel also has a high nerve induction effect [47,48]. In a mouse tibial nerve model, keratin hydrogel significantly improved the electrophysiological recovery in the early stage. At 6 months, the keratin hydrogel group even produced electrical and histological results superior to the empty conduits and sensory nerve autografts [49]. In 1 cm rat sciatic nerve injury, keratin hydrogel could promote debris clearance in the distal stump by Schwann cell activation [50].

Chitosan is formed by the deacetylation of chitin in crustacean shells. It is a widely used tissue engineering material with a low production cost. It has the potential to interact with regeneration-related cells and neural microenvironments, so as to improve axon regeneration and reduce the formation of neuroma. Chitosan can effectively maintain the biological activity of loaded cells and promote their proliferation and differentiation [51], such as promoting the growth of Schwann cells [52], supporting the differentiation of neural progenitor cells [53], and inducing mesenchymal stromal cells to differentiate into Schwann-like cells [54]. It allows a functional and morphological nerve regeneration similar to autologous nerve transplantation [55]. Jafar prepared chitosan scaffolds containing human endometrial stem cells and platelet plasma. It promoted nerve fiber regeneration and angiogenesis and prevented the formation of scar tissue in a rat spinal cord injury model [56]. In addition, chitosan oligosaccharide, the degradation product of chitosan, showed a neuroprotective effect in the process of peripheral nerve regeneration. It could promote cell proliferation, prevent cell apoptosis and accelerate peripheral nerve regeneration [57,58,59].

Alginate is a polysaccharide found in brown algae. It has an antioxidant capacity and can protect nerve cells from H_2_O_2_ damage [60,61]. Alginate hydrogel can not only promote the adhesion and proliferation of nerve cells [62], but also enhance the differentiation of pluripotent cells (mouse embryonic stem cells) into neural lineages [63]. It can also trigger the regeneration of up and down axon fibers and effectively form synapses, which play a supporting role in axon regeneration [64]. However, alginate hydrogel still has some limitations. Since mammals do not produce endogenous seaweed enzymes, the alginate hydrogel implanted in vivo may take several months to complete degradation. This poor degradation will reduce the penetration ability of cells [65]. In addition, pure alginate hydrogel is bioinert and cannot promote the activity of encapsulated cells [66]. For its applications, it can be improved by compounding with other materials [67], or adding substances such as nanofibers [68]. 

Silk fibroin and sericin are two main proteins in silk. Sericin has neurotrophic and neuroprotective effects, can promote axon extension and branching, and prevent the cell death of primary neurons in a hypoxia environment [69,70]. Sericin hydrogel can promote the adhesion and differentiation of neurons and facilitate the repair of nerves [71]. In addition, the degradation products of sericin protein are rich in glycine and serine, which play an important role as neurotransmitters in neurotransmission [72]. Silk fibroin has excellent nerve biocompatibility and can support nerve regeneration. It has a high potential in nerve tissue engineering. At present, it is mainly used as a nerve conduit to bridge damaged nerves by electrospinning [73,74,75]. Silk fibroin hydrogel has good biocompatibility with ganglion, and is beneficial for maintaining the activity of Schwann cells [76], promoting axonal growth and guiding axonal sprouting [77].

### 2.2. Synthetic Biomaterials

Compared with natural-based biomaterials, synthetic biomaterials refer to materials processed by chemical methods or the polymerization of different substances, which have the advantages of mass production, low cost, stable properties, no immune sources, and so on. They are also widely used in nerve repair. 

Polyethylene glycol (PEG) is a stable, nontoxic and biocompatible polymer synthetic material with a wide range of clinical applications. When PEG is used for spinal cord repair, it has many advantages, such as no accumulation in the body, inhibiting the formation of vacuoles and scars, reducing inflammation and so on [78]. PEG can be used to synthesize hydrogels and modify other hydrogels [79]. When repairing sciatic nerve transection injury in rats, PEG hydrogels can effectively reduce scar tissue formation [80], support axon regeneration [81], and promote limb function by promoting more and more myelin regeneration, enabling a faster recovery [82]. Myelin refers to the protective shell formed by oligodendrocytes on the outside of nerve fibers. Its main function is to protect nerves and participate in the conduction of nerve impulses. Changing the end groups of PEG and introducing different functional groups, such as toluene sulfonate, amino group, carboxyl group and aldehyde group, can further expand the application field of PEG. By introducing biological ingredients, such as natural biomaterials and nutritional factors, into synthetic hydrogels, PEG can effectively improve the function of nerve cells in inert PEG hydrogels, and promote the survival and growth of nerve cells [83,84,85]. Zhang prepared hydrogels made from graphene oxide and four-arm polyethylene glycol terminated with two acetyl glycerol and loaded with the anti-inflammatory agent, diacerein. The hydrogel was delivered to the spinal cord in rats, causing injury by a minimally invasive injection. Free diacerein can minimize inflammatory responses and prevent the formation of an inhibitory microenvironment. Graphene oxide gives proper conductivity to hydrogels, which promotes the neuronal growth and remyelination of axons [86]. 

Methacrylate-based hydrogels are widely used in the field of nerve repair. Hydrogels based on poly(2-hydroxyethyl methacrylate) (PHEMA) and poly[N-(2-hydroxypropyl)-methacrylamide] (PHPMA) belong to a group of synthetic, highly biocompatible polymers [87]. The high water content of the PHEMA hydrogel and its mechanical compatibility with host tissues allows it to restore the anatomical continuity of damaged nerve structures. Porous PHEMA hydrogels inhibit scarring [88] and provide scaffolds for the growth of connective tissue elements, blood vessels, neurofilaments, and Schwann cells [89]. In addition, it can also be used as a platform for continuous drug delivery. The PHEMA also has some disadvantages. First, as a non-degradable polymer, hydrogel calcification and prolonged inflammatory responses might limit long-term axonal regeneration [90]. Biodegradable hydrogels were prepared by compounding PHEMA with other polymers. Pertici combined PHEMA with PLA to obtain block copolymer. The PLA-b-PHEMA block copolymer can indeed display desired properties, such as biodegradability and an improvement in rat motor function, which were observed after implantation following a thoracic spinal cord hemisection in rats [91]. In addition, PHEMA itself is not adhesive or attractive to neurons, so it can be modified using substances with biorecognition sites, thereby improving the biocompatibility of the hydrogels. Šárka used laminin-derived peptide sequences to modify PHEMA to promote cell adhesion and neural differentiation [92]. The modification of PHEMA hydrogels with lysine can prolong the release time of its loading factor, increase the survival rate of neurons, and induce favorable biological reactions [93].

PHPMA is more biocompatible than PHEMA [94]. The PHPMA hydrogel implantation into the hemisected T10 rat spinal cord induced locomotor and neurophysiological improvements [95]. Six months after the PHPMA hydrogel was implanted into the spinal cord of transected adult cats, the interface formed between the hydrogel and the spinal stumps prevented scar formation. The presence of the hydrogel implant led to a considerable reduction in damage to distal caudal portions of the spine and provided an adequate environment for growth of myelinated fibers [96]. Peptides- and aminosugar-modified PHPMA hydrogels could increase adhesion properties with host neural tissue, promote the migration and reorganization of local wound repair cells, and be filtrated by host nonneuronal cells [97]. Woerly prepared a PHPMA hydrogel with a bulk-modified saccharidic portion of ganglioside GM3 (The 3′-sialyllactose is a bioactive epitope recognized by many cell surface receptors on cells). The modified hydrogel was well-tolerated by the neural tissue and had the capacity to store endogenous self-migrating stem cells and, for a proportion of them, to support their neural differentiation in a non-neurogenic region [98]. Similar to PHEMA, PHPMA is also a non-degradable polymer, which can conjugate with degradable polymers to expand its application range [99].

Polyvinyl alcohol (PVA) is a crystalline polymer prepared from polyvinyl acetate. Although PVA can be hydrolyzed, it cannot be completely degraded, which is the main disadvantage limiting its application in biomedical fields. PVA conduit does not support the adhesion and proliferation of human neuroblastoma cells in vitro [100]. However, in vivo experiments showed that PVA hydrogel prevented the migration of inflammatory cells after the laminectomy of cats. At the same time, it reduced the formation of scar tissue [101]. Heang synthesized PVA/HA hydrogels, which can promote the differentiation of human-bone-marrow mesenchymal stem cells into specific cell types by adjusting the hardness of hydrogels [102]. 

In short, natural-based biomaterials possess an excellent biocompatibility and some may have a bioactive sequence in the moiety, which can benefit nerve repair to some extent. However, there are slight differences in the performances of natural-based biomaterials between different batches as they are extracted from animals and plants, limiting their further translation into the clinic. Thus, it is important for natural-based biomaterials to pay attention to maintaining performance uniformity. Synthetic biomaterials have a better performance uniformity, but they lack biocompatibility and a bioactive sequence. Thus, it is necessary for synthetic biomaterials to be modified with some natural molecules to improve their biocompatibility when they are applied in nerve repair. Additionally, it is also vital that the degradable product of synthetic biomaterials should be bio-safe.

## 3. Hydrogels as Carriers for Nerve Repair

### 3.1. Cell Carrier

As an ideal carrier system for nerve repair, hydrogels have a good biocompatibility and mechanical strength, which can transport multiple cells and release regulatory factors over a long time period in damaged parts [37]. Currently, cell transplantation is considered to be one of the most promising methods in nerve repair. Neural progenitor/stem cells, mesenchymal stem cells and Schwann cells, etc., are widely used in nerve repair [103].

**Figure 3:**
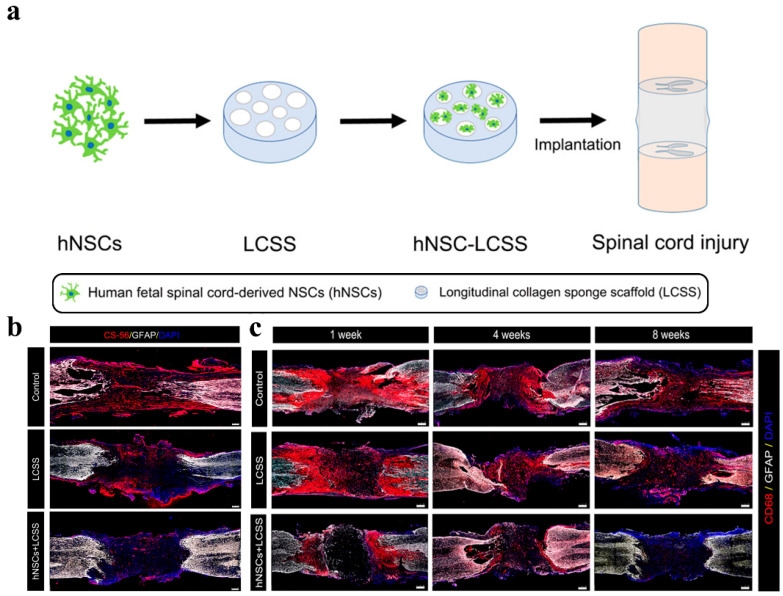
Schematic diagram and repair effect of neural stem cells in spinal cord repair. (**a**) Scheme of neural stem cells implantation for spinal cord repair; (**b**) Images of CS-56 immunostaining representing the formation of glial scars in the injury cavity at 8 weeks post-operation. The hNSCs implantation efficiently reduced glial scar forming around the injury cavity; (**c**) Representative immunofluorescence images of CD68 (activated microglia cell and macrophage marker)-positive signals in damaged sites. Grafted neural stem cells significantly suppress inflammation. (Adapted with permission from Ref. [106]. 2020, American Chemical Society).

Stem cells are a kind of cell that can self-renew and differentiate into a variety of cells. The main goals of stem cell therapy are to regenerate axons, prevent apoptosis and replace lost cells. Neural progenitor/stem cells were the first stem cells to show immunomodulatory properties and one of the most studied and promising stem cell subtypes [104]. Nowadays, the results of in vivo studies indicate that, in nerve injury models of zebrafish embryos, hydrogels can significantly promote nerve growth by introducing neural progenitor cells/stem cells [105]. Moreover, the use of hydrogels to transport neural stem cells can also effectively improve the survival rate of these cells. At the same time, neural stem cells are more evenly distributed in the damaged nerve area. As shown in Figure 3, Zou transplanted collagen scaffolds loaded with human federal spinal-cord-derived neural stem cells into rat spinal cord transection injury. It was found that neural progenitor cells/stem cells also have a stronger neural differentiation tendency and can effectively reduce the formation of a glial scar and inflammation in Figure 3b [106]. Zhang expanded the application of nonionic and thermoresponsive diblock copolypeptide hydrogels as vehicles for transplanting cell suspensions into the central nervous system. Hydrogels support the long-term viability of suspended cells in vitro, and their viscosity can prevent cell sedimentation. The thermoresponsiveness of hydrogels allows for neural stem cell suspensions to be easily injected as liquids at room temperature. At body temperature, liquids self-assembled into hydrogels with a stiffness tuned to that of central nervous system tissue. Compared with the direct injection of neural stem cells into the central nervous system through small-bore cannulae, hydrogels significantly increased the survival of neural stem cells grafted into a healthy central nervous system by threefold. Neural stem cells, injected into hydrogels of the injured central nervous system of mice, gave rise to new neural cells that distributed throughout large tissue lesions and supported the regrowth of host nerve fibers into and through lesion core tissues, which were devoid of host neural cells [107]. Neural progenitor cells have been shown to promote cell-contact-mediated axon regeneration [108].

Mesenchymal stem cells are also considered to be an important cell source for nerve repair after nervous system injury. Mesenchymal stem cells have strong proliferative ability, multi-directional differentiation potential and immunomodulatory ability [104]. They are also suitable for solving the physiological and pathological problems caused by nerve damage and have gradually become the dominant cells used to repair nerve function [109]. Mesenchymal stem cells include bone-marrow stromal cells [110], adipose-tissue-derived mesenchymal cells [111], human-umbilical-cord-blood-derived mesenchymal stem cells [112], etc., which can differentiate into Schwann-like cells to replace damaged cells and rebuild neural circuits, secrete cytokines to provide neuroprotection and nutritional support for injured nerve and axon regeneration, and promote the regeneration of neurons. Mesenchymal-stem-cell-derived factors can affect the maturation and function of all-immune cells and suppress both innate and adaptive immunity [113]. He found that mesenchymal-stem-cell-spheroids-loaded collagen hydrogels possessed both a superior anti-inflammatory efficacy and neurogenic activity. Mesenchymal stem cells spheroids promote cell–cell interactions and cell communication, accelerate the secretion of endogenous nutritional factors and the extracellular matrix, and facilitate the differentiation of neural stem cells into neuronal lines. In addition, the formation of the mesenchymal stem cells spheroids secreted more amounts and types of cytokines, as well as immunomodulatory paracrine factors to suppress lipopolysaccharide-induced inflammatory reactions [114]. Dental pulp stem cells are bone-marrow mesenchymal stem cells derived from dental pulp. Because they will not cause additional harm to the body or cause ethical problems due to the source of the cells, dental pulp stem cells have also attracted extensive attention. Luo selected the 10% formula (10% gelatin methacrylate hydrogel, recombinant human basic fibroblast growth factor and dental pulp stem cells) to fill a cellulose/soy protein isolate composite membrane tube to construct a nerve regeneration conduit. This conduit was applied to repair a 15 mm long defect of the sciatic nerve in a rat model. After 12-week post-implant surgery, it was found that almost all newly formed nerve tissues at the defect site originated from the direct differentiation of exogeneous dental pulp stem cells [115]. Dental pulp stem cells have a positive effect on nerve regeneration through glial differentiation and neuroprotective/neurotrophic effects on host tissues and significantly promote axon regeneration [116,117].

Schwann cells are the main glial cells of the peripheral nervous system and an important part of endogenous repair. Schwann cells form the myelin surrounding the nerve fibers and create a structural support to the axons. They also secrete growth factors essential for the maintenance of neuronal cells and provide directional clues to the regrowing axons [118]. Mosahebi encapsulated Schwann cells in different hydrogel materials. The viability and growth of Schwann cells were different, but there was no significant difference between these Schwann cells and those cultured on culture plates. Compared with alginate hydrogels without the encapsulation of Schwann cells, hydrogels-encapsulated Schwann cells significantly enhanced the mean neurite extension and increased the number of neurites sprouts of the chick embryo dorsal root ganglia [119]. In the collagen- and hyaluronic-acid-interpenetrating polymer network hydrogels prepared by Suri, Schwann cells with a high cell density in the hydrogel structure could actively secrete the nerve growth factor and brain-derived neurotrophic factor. In the co-culture of dissociated neurons and Schwann cells, some neurites were also observed to extend following the introduction of Schwann cells [120]. Compared with the use of gelatin sponge blocks to transplant Schwann cells suspension to the injury site, the transplantation of Schwann cells seeded in alginic acid hydrogel to the injury site of the rat spinal cord could inhibit cellular apoptosis, and the motor function of rats recovered better after 21 days [121]. However, it should be noted that, compared with homogeneous Schwann cells, heterologous Schwann cells elicit a strong immune reaction and hinder nerve regeneration when transplanted to the damaged site [122]. This suggests that researchers should pay attention to immunosuppression when using Schwann cells and other cells that can cause an immune response to nerve repair, or use autologous cells to obtain a better repair effect.

### 3.2. Bioactive Compounds

In order to avoid the problems of cell survival and a capacity decline, immune rejection, and limited cells, a number of studies have directly loaded cell components and cell secretions into hydrogels, which promote functional recovery from different aspects [123].

Exosomes are nano-scale extracellular vesicles and natural carriers. Their phospholipid bilayer structure can protect biologically active substances while loading growth factors, RNA, small-molecule drugs, etc., and achieve sustained release. Due to their nano size, exosomes can not only escape phagocytes and freely shuttle between cells or matrixes, but they also have a strong penetrating ability. Mesenchymal stem cells are the main source of exosomes for tissue repair. Exosomes contain specific substances from parental cells. The engineered preparation of exosomes can be achieved by the targeted modifications or genetic modifications of parental cells. As a cell-free biomaterial, exosomes can partially solve the problems encountered in the clinical application of regenerative medicine, such as the source, quantity and immune response of seed cells [124]. Li encapsulated exosomes derived from mesenchymal stem cells in a peptide-modified adhesive HA hydrogels, and the implanted exosomes exhibited an efficient retention and sustained release in the host nerve tissues. In the rat spinal cord injury model, hydrogels elicit significant nerve recovery and tissue preservation by effectively mitigating inflammation and oxidation [125]. Yang encapsulated Neurotrophin-3 mRNA in adipose-derived stem cell-derived exosomes. The exosomes were loaded into the alginate hydrogel and subsequently loaded into the nerve guide conduit to bridge sciatic nerve defects in rats. The exosomes could stably release Neurotrophin-3 mRNA for at least 2 weeks [126].

Some biologically active factors can regulate and promote nerve repair by interacting with corresponding receptors. At present, many studies have reported that various nutritional factors and growth factors can promote the survival and growth of neurons, and the therapeutic effect of active factors in nervous system injury has attracted more and more attention. However, active factors usually suffer from a reduced bioavailability, poor tissue distribution, short half-lives and so on. Higher doses may cause adverse immune reactions. Therefore, combining active factors with tissue engineering materials, especially hydrogels, can maintain their activity for a long time, reduce the impact of the blood–brain barrier, directly release active factors at the injury site, and exert better therapeutic effects.

Neurotrophins are a family of growth factors that play important roles in the survival and function of neurons. The mammalian neurotrophic factor family has four known members: the brain-derived neurotrophic factor (BDNF), nerve growth factor (NGF), neurotrophin-3 (NT-3) and neurotrophin-4 (NT-4). Neurotrophins can regulate the development of the central and peripheral nervous system and inhibit apoptosis. Neurotrophins are key mediators that promote neuronal growth and survival after neuroplastic injury. Currently, the first two neurotrophic factors are under more intensive research as potential stroke treatments [127]. Cook utilized BDNF-loaded hyaluronic-acid hydrogels to locally release BDNF in the infarct cavity of a mouse stroke model for neural repair in the chronic phase after stroke. The experimental results showed that, compared with the direct injection of BDNF, the diffusion time of hydrogel-delivered BDNF from the stroke cavity to the surrounding infarct tissue was prolonged, and the recovery of motor function was promoted. Hydrogel–BDNF induces the initial migration of immature neurons into the peri-infarct cortex and enables long-term survival [128]. While loading NGF in the hydrogel to achieve a long-term effective release, the resulting nutritional factor gradient can stimulate the directional neurite extension of the ganglion in the hydrogel [129]. The hydrogel load with NGF exhibited a good morphology and stable bioactivity in vitro in Zhao’s research. The cell uptake efficiency of NGF was significantly improved. Tissue repairment in spinal gray matter was observed, including the recovery of nuclei and morphology, as well as a reduction in the organization cavity in the NGF–hydrogel group. Hydrogels could maximize NGF’s effects on neuron protection and spinal cord injury recovery [130].

The angiogenesis and reconstruction of a functional microvasculature is an important and necessary step to promote the recovery of nerve injury. The vascular endothelial growth factor (VEGF) has beneficial effects on motor neuron survival, neuronal viability and proliferation. VEGF is expressed in the nervous system after injury and participates in the process of nerve repair by regulating angiogenesis, neurogenesis, and neurite outgrowth through signal transduction [131]. Gelatin hydrogels loaded with VEGF can induce capillary-like tube formations and axonal outgrowth in vitro [132]. When the 15 mm rat sciatic nerve defects were repaired by a chitosan hydrogel loaded with a neurotrophic factor and VEGF at the same time, it can be observed that the hydrogel not only promoted nerve regeneration, but also promoted blood vessel penetration in the early stage of injury [133].

In addition, fibroblast growth factors (FGF) are also beneficial for neural repair, some members of which are produced by neurons and glial cells, in turn targeting other cells. Basic fibroblast growth factor (bFGF), a widely studied member of the FGF family, is abundant in the central nervous system. bFGF is closely related to neuroprotection, injury repair, and neurogenesis, and is an important contributor to the plasticity of hippocampal neurons. Fujimaki repaired a 15 mm sciatic nerve defect in a rat using a bFGF-loaded oriented collagen hydrogel tubes. The results indicated that bFGF could promote the regeneration of more myelinated fibers and the improvement of motor function [134]. Zhang used bFGF gene-modified neural stem cells to improve neurological deficits after transient middle cerebral artery occlusion in rats. The transplanted modified cells improved survival, migration, proliferation and differentiation in the brain. Moreover, bFGF promoted the differentiation of modified cells into mature neurons within the infarcted area [135]. Similar to bFGF, the acidic fibroblast growth factor (aFGF) can also regulate cell proliferation, migration, differentiation and survival, and down-regulate known axonal regeneration inhibitors, which play an important role in the regeneration of nerve fibers. After filling the collagen hydrogel with aFGF, it showed an effect equivalent to that of autologous transplantation, which had a significantly better regenerative ability than other groups (the empty conduit and the nerve conduit containing hydrogel only) in repairing a 10 mm defect of the rat sciatic nerve [136].

In addition, drugs can also be loaded into hydrogels to achieve long-term release. For example, when anti-inflammatory drugs and growth/nutrition factors are simultaneously loaded in hydrogels, the coordinated release of multiple components can effectively protect spare tissues/axons from secondary damage, reduce the expression of proinflammatory cytokines, cystic cavitation and glial scar formation and improve the survival rate of neurons [137,138]. The active ingredient of traditional Chinese medicine is also loaded into a hydrogel for nerve repair. Berberine is a plant alkaloid-based substrate that can promote the growth, development and reconstruction of axons in damaged peripheral nerves and the central nervous system. It has a neuroprotective effect on damaged neurons. Sadeghi loaded berberine in chitosan nanoparticles within a hybrid of alginate and chitosan hydrogel. The scaffolds with endometrial stem cells, as well as scaffolds alone, were transplanted into hemisected spinal cord injury rats. The data from the immunostaining of a neurofilament, as a neuronal growth marker, showed that all scaffolds had a certain immunoreactivity [139]. Curcumin is a diketone compound with an antioxidant activity and anti-inflammatory effect. Qian injected curcumin-loaded hydrogel under the endocranium into the traumatic brain injury mice. The slow and sustained released of curcumin can further reduce the level of reactive oxygen species in traumatic brain injury tissues and brain edema [140]. In Luo’s research, Curcumin encapsulated in the hybrid hydrogel was released in a sustained pattern over a 7-day period. The presented hybrid hydrogel could easily reassemble the extracellular matrix locally at the lesion site of rat spinal cord, and further modulate local inflammatory reaction by regulating the phenotypes of infiltrated inflammatory cells [141].

Undoubtedly, the strong loading characteristics of hydrogels make them a universal carrier for all the scenarios, including brain, spinal cord and peripheral nerves. Next, they also make the design of a complex formulation possible, with multiple components that play different roles in nerve repair, which is thought to be more powerful for repairing nerves (Dai’s paper). Furthermore, how to balance the physiological environmental conditions and the physicochemical properties of hydrogels, so as to precisely regulate the physical and chemical parameters of damaged nerve systems to better promote nerve repair, is the most important direction that needs to be studied.

## 4. Fabrication and Processing of Hydrogels for Nerve Repair

**Figure 4:**
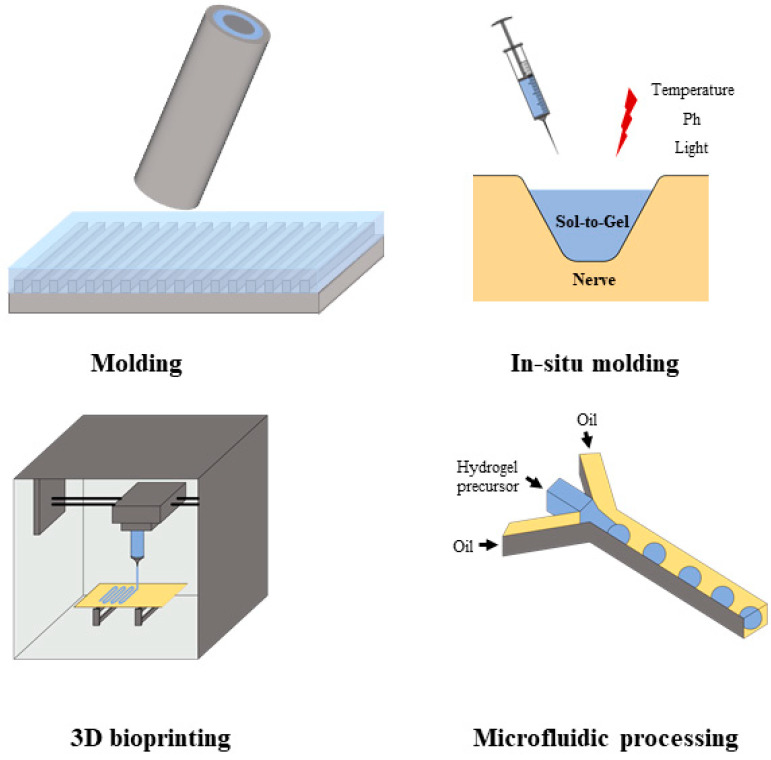
The types of preparation and processing of hydrogels for nerve repair.

Different scenarios of nerve repair require hydrogel carriers with specific physical shapes. Brain injury, such as the neural cavities induced by stroke, may have an arbitrary shape. The hydrogel carriers should form a matching shape in vivo. On the other hand, the shape of the conduit is more conducive to bridging and repairing the spinal cord and peripheral nerves. Therefore, shape modulation has become one of the most important problems in the preparation and processing of hydrogel carriers. This paper mainly explores four fabrication and processing methods of hydrogels, as shown in Figure 4.

### 4.1. Template Method

The template method is to solidify the gel by depositing a hydrogel precursor fluid on the mold, causing in situ gelation. The template method has the advantages of a repeated mold operation, simple operation, and high repeatability, and can produce a large number of hydrogel carriers with a high uniformity in a short time. The template method is the most commonly used method for preparing nerve conduit by hydrogels. Hydrogels can be directly solidified into hydrogel conduits with ideal diameters and lengths by using mold materials that are easy to separate from hydrogels, such as silica gel, polystyrene, poly methylsiloxane, and so on [142]. Itai demonstrated a two-layer conduit loaded with Schwann cells, which was prepared by means of an improved template size and preparation sequence. The conduit was composed of two coaxial hydrogel layers of chitosan, and the collagen was made via molding and a mechanical anchoring attachment with holes made on the hydrogel tube [143]. In addition, the template method is less restricted by the mechanism of gelation and is suitable for the preparation of various hydrogels. Ma used high-purity iron wire as the starting template. Hydrogel tubes with adjustable size, mechanical strength, good elasticity and strong bearing properties were prepared by free radical polymerization on the surface of the wire. After two crosslinking treatments, the hydrogel layer could be easily stripped from the wire. This method can be used to design a series of complex 3D hydrogel tube arrays, hollow closed hydrogel units and hydrogel tubes embedded in functional material [144].

The template method is the most convenient way to change the surface morphology of hydrogels. By changing the macro-shape or microtube morphology of the mold, the surface morphology of the hydrogel can be indirectly regulated, so that the surface of the hydrogel has different morphologies such as cavities, grooves and ridges. The hydrogel provides the terrain clue for subsequent cell cultivation, increment and extension [77]. Li prepared an anisotropic polyacrylamide hydrogel with an aligned ridge structure by in situ free radical polymerization and PDMS micromolding, so that the hydrogel surface has a sharp fringe pattern [145]. Shahriari used a fiber mold to introduce a linear microchannel inside the alginate hydrogel, and the multi cavity alginate hydrogel was prepared. In vitro experiments demonstrated that the scaffold could maintain its channel and volume geometry for at least 28 days [146].

### 4.2. In Situ Injection Molding

In situ injection molding hydrogel has the ability of rapid prototyping. The advantage of in situ injection molding is its high plasticity, which can flexibly match any specific size and shape of damaged cavity. The hydrogel precursor was directly injected into the cavity in first step. It was easy for the injected solution to fill the cavity with an arbitrary shape. Then, it changed from solution state to gel state by internal/external stimuli such as light, heat and pH conditions. Injection allows hydrogels to transmit in vivo in a minimally invasive way to facilitate surgery, while allowing patients to have less pain and a smaller scar size, and thus recover faster [147].

**Figure 5:**
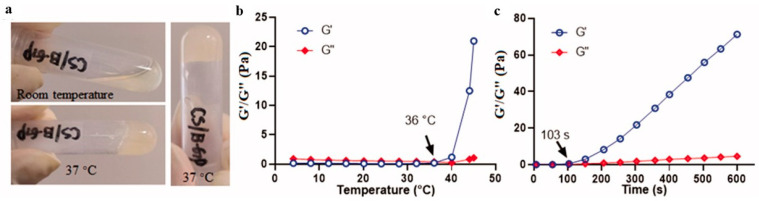
Formation of thermosensitive hydrogels (**a**) The representative images of thermosensitive hydrogels; (**b**) The temperature-dependent changes in the modulus of the hydrogel; (**c**) The time-dependent changes in the modulus of the hydrogel at 37 °C. (Reprinted with permission from Ref. [148]. 2020, Taylor & Francis).

As shown in Figure 5, Wang developed a thermosensitive hydrogel based on chitosan. This composite hydrogel can undergo a sol–gel transition within a few minutes within the physiological pH value and at 37 °C. The hydrogel is used for the local and sustainable transport of neurotrophic factors and immunosuppressants to the nerve. The in vivo experiment of optic nerve injury in white rabbits showed that the drug could be continuously released for 9 days. It had a protective effect on traumatic optic nerve injury with adverse reactions [148]. Zhao synthesized a new heparin-poloxamer thermo-sensitive hydrogel with a controllable phase transition temperature suitable for in situ administration. In characteristic experiments, the hydrogel was liquid at room temperature (4 °C). At a normal temperature (37 °C), the gel could be transformed into a three-dimensional network structure. Compared with the direct injection of nerve growth factor solution, hydrogels injected in situ can be locally transported to the injured spinal cord of rats to enhance the uptake efficiency of the nerve growth factor by rats in the spinal cord injury [130]. As a delivery carrier, hydrogels not only have a good affinity for a large number of growth factors, but can also control their release in a stable way [149]. Zhang prepared a pH-induced chitosan HA hydrogel. The gel solution could undergo rapid gelation at 3 min when the environmental pH value changed to 7.4. The hydrogel has a porosity of about 80% and a good swelling behavior. It can be reduced by about 70% in 8 weeks, which is suitable for the sustained release of growth factors [150]. Theoretically, photoinitiation is also an important mechanism for the rapid gelation of injectable hydrogels in vivo. However, considering the irreversible damage to the human body caused by UV light and the potential toxicity of photoinitiators, the practical application of a UV-cured hydrogel system is limited. In contrast, near-infrared light (NIR) and other long wavelengths of light are considered safe for cells. Therefore, it is highly desired to develop an NIR-induced hydrogel for nerve repair.

### 4.3. 3D Bioprinting

Due to its precise control in the manufacturing process and flexibility of design, 3D bioprinting technology has become a powerful material preparation technology. The process of 3D bioprinting creates a suitable microenvironment for the regeneration of damaged tissues by depositing biological ink layer-by-layer to reproduce the inherent cell composition and tissue structure [151]. Hydrogels with adjustable physical properties and great biocompatibility can not only serve as a structural scaffold for printing tissues, but also provide a microenvironment for cell packaging and guide cell activity, thus becoming the most promising candidates in biologic ink. Printed neural-like in vitro constructs showed a uniform cell growth and significant biological characteristics [152]. Through 3D bioprinting, we can directly build multi-channel hydrogels and use them as a nerve conduit [153]. Xu rapidly customized a hydrogel nerve conduit with a different internal diameter, wall thickness and microfiber by using a continuous 3D bioprinting process based on digital light processing. The drug-loaded nanoparticles were evenly distributed in the conduit. An ideal compression performance means that the conduit can maintain structural integrity during implantation [154]. In addition, 3D bioprinting can also be used for the preparation of hydrogel microstructures. Yoo prepared oriented collagen hydrogels as a filler for electrospinning poly (lactic acid) caprolactone (PLCL) elastomer to achieve a microscale hydrogel pattern. Based on sciatic nerve injury models in rats, these hydrogels confirmed the beneficial effects of the nerve-guided conduit with 3D bioprinted collagen hydrogel on axonal regeneration and remyelination along with superior functional recovery in comparison with the conduit filled with the bulk collagen hydrogel [26]. The process of 3D bioprinting can support various cell types and active factors in the nervous system. This helps us to create more complex bionic neural tissues [155,156]. Li encapsulated Schwann cells in the compound sodium alginate gelatin gel solution, and printed through the extruded biological printer to build a customized shape of the three-dimensional hydrogel structure. Printed hydrogels supported Schwann cell proliferation for 2 weeks. After 14 days of culture, Schwann cells cultured in printed structures maintained a viability of 92.34 ± 2.19% and showed an enhanced capability of NGF release (142.41 ± 8.99 pg/mL) compared with cells from two-dimensional cultures (92.27 ± 9.30 pg/mL) [157]. By adjusting the printing parameters of hydrogels, such as the bioprinting speed and crosslinking conditions, they can also indirectly regulate the growth characteristics of loaded cells [158].

The ideal neural scaffold requires not only reliable mechanical properties but also an elaborate guiding structure, which is well-addressed by the highly controlled flexible 3D bioprinting methods [159]. Through 3D bioprinting technology, we can not only achieve a more refined design of the macro structure and micro morphology of hydrogel, but also make hydrogel materials more widely applied in the field of nerve repair. It is also one of the most promising means to realize nerve “customized” repair since printing parameters can be flexibly adjusted according to needs.

### 4.4. Microfluidic Processing

Microfluidic technology is a new technology used to accurately control and manipulate micron-scale fluids on microchips. Microfluidic technology has great potential in chemistry, biology, medicine and other fields with its advantages of miniaturization and integration. Microfluidic technology can be used to prepare hydrogel fibers with a uniform diameter and adjustable mechanical strength [160], as well as different lengths [161]. Zhao proposed a microfluidic spinning system for flexibly generating grooved microfibers relying on the volume change after ionic crosslinking of sodium alginate with different concentrations. The size and shape of the fibers are tuned by the viscosity and concentration of the solution, as well as the flow rates, in a controllable manner. The cells grown on the microfibers exhibited an ordered alignment [162]. Jung prepared PU microfibers that used laminar flows of multiple streams in a microfluidic system. The porous region that was spontaneously formed by carbon dioxide bubbles on the microfiber could promote cell adhesion. Microfibrils showed significant improvements in cell proliferation and viability [163]. Alginate hydrogel microfibers with a highly complex cross-sectional morphology were prepared by Yoichi Kitagawa. Neurons such as PC12 cells were encapsulated in the parallel region, which was made of a soft hydrogel matrix. Cells proliferated along the fiber lengths because of physical restrictions imposed by the relatively rigid regions. After 14 days of culture, the microfibrils showed a linear intercellular network similar to the structure of nerve bundles in vivo. By adjusting the structure of the microfluidic chip and the flow rate of the precursor solution, the microfiber could have a freely controllable cross-sectional morphology [164]. Chen used the microfluidic system to construct a methacrylic acid/HA hydrogel microfiber with s core shell structure and analog neural structure. Neurons and Schwann cells were located in the core and shell of gel fibers, respectively. Compared with being encapsulated in single-layer fibers, the cells showed better neural properties, such as promoting neuronal arrangement and elongation and Schwann cell myelin maturation [165]. Wu made a collagen/poly-pyrrole nanoparticle mixed hydrogel microfiber using a microfluidic chip. The oriented fiber microstructure enhanced neuron-like cells aligned with fiber axons [166].

Microfluidics technology is also an ideal platform for constructing hydrogel microspheres and simultaneously loading drugs and cells. In the microfluidic chip, two incompatible liquids enter different microchannels as continuous-phase and dispersed-phase liquids at different liquid flow rates, respectively. When the two fluids meet at the intersection, the dispersed phase fluid continues to extend to form a “spherical” or “jet” liquid column, and then under the shear and extrusion of the continuous phase fluid. Due to the instability of the free interface, the dispersed phase disperses in the continuous phase in the form of small-volume units to form droplets. The hydrogel microspheres can be formed under the corresponding gelation conditions [152,167]. The main advantage of microfluidics using microfluidics is that the size of microgels can be precisely controlled by adjusting the chip structure and the ratio of the two-phase flow rate [168]. The resulting hydrogel microsphere has a good dispersibility and stability. Additionally, it requires less sample and solvent consumption and is suitable for very low concentration samples [169].

**Figure 6:**
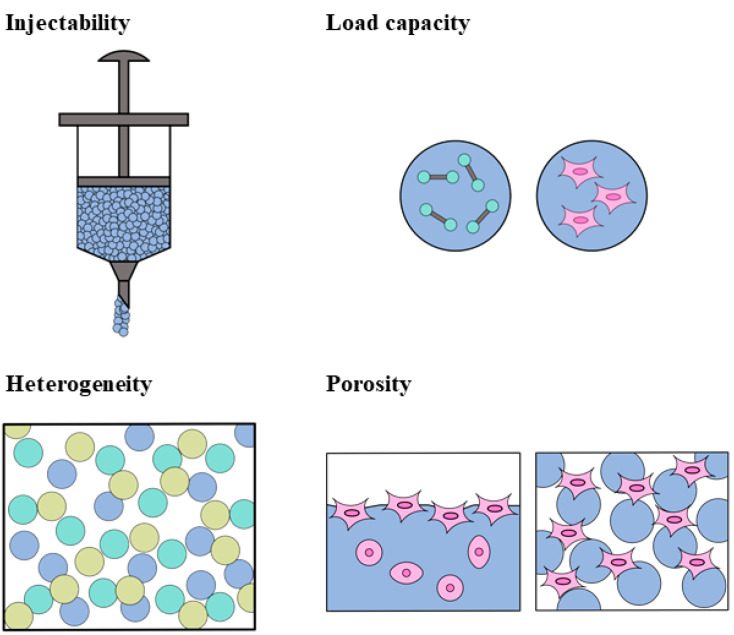
Advantages of hydrogel microspheres for nerve repair.

With the development and application of the hydrogel microsphere, researchers have paid increasing attention toward using microspheres to repair damaged nervous systems [170]. In practical applications, bulky hydrogel may not accurately meet the expected requirements. For example, in brain injury, traumatic defects are usually small and irregular in shape. Since bulky hydrogels often cannot be filled perfectly, a hydrogel microsphere is an alternative approach in this case. Hydrogel microspheres have many advantages in practical applications over traditional bulky gel. First, they are another injectable hydrogel, which can be injected through a needle in a gel state by virtue of the shear-thinning effect. Second, hydrogel microspheres containing different active substances and different sizes can be mixed to obtain multiple functions [171]. Third, the hydrogel microspheres possessed a higher porosity, as shown in Figure 6, which is beneficial to cell growth and proliferation. Gelatin methacryl/chitosan microspheres were prepared by microfluidic method by Chen. The microspheres were round, with a uniform size and relatively smooth surface. They have mechanical properties suitable for nerve cell proliferation and differentiation. In vitro experiments show that microspheres have a good biocompatibility and provide a surface microenvironment suitable for the adhesion and growth of PC12 cells. The loaded nerve growth factor can further induce axon growth and the elongation of PC12 cells, while maintaining their biological activity, which makes it suitable as a cell carrier [172,173]. Microspheres can not only load various growth factors, but also load all kinds of nerve cells. Alessandri loaded human neural stem cells using a hollow hydrogel. The results show that cells can further differentiate into neurons in the capsule [174].

**Figure 7:**
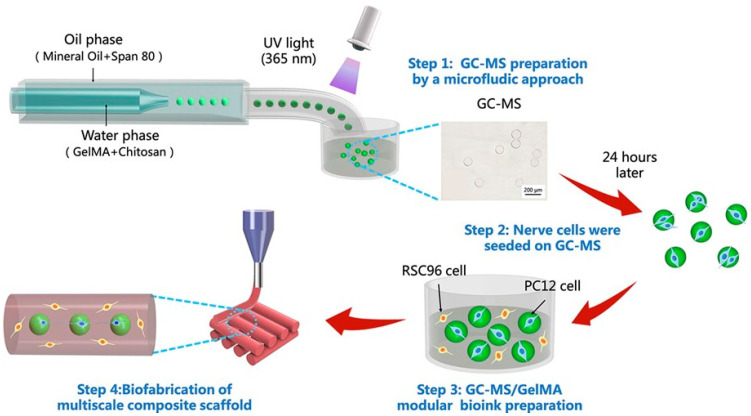
Flowcharts of microfluidic and 3D printing for the preparation of hydrogel neural scaffolds. (Reprinted with permission from Ref. [172]. 2020, Elsevier).

Although in situ injection, which injects solution into the brain cavity, and in situ gelation can also form a matchable bulky gel to fit the cavity, this requires a precise control of gelation time. On the one hand, if the gelation time is too short, the gel solution will complete the liquid–solid transformation before it is fully delivered to the cavity. On the other hand, if the gelation time is too long, the gel solution may diffuse into the surrounding tissues before gelation. Thus, the injectable hydrogel microspheres are more convenient for practical operations. Furthermore, hydrogel microspheres can also be used as biological ink in 3D printing technology [175]. The technology of 3D printing allows for the use of various forms of hydrogel microspheres systems [176]. As shown in Figure 7, hydrogel microspheres loaded with nerve growth factors were mixed with gelatin methacryl solution, and a multi-scale composite hydrogel scaffold was constructed by 3D printing. It provided a 3D macro environment for Schwann cell proliferation and nerve cell organization and played a better protective role [172]. By using hydrogel microspheres to further encapsulate growth factors, the release rate of growth factors can be reduced and the activity time in vivo can be prolonged [177,178].

In summary, the different fabrication and processing techniques endow hydrogel with a powerful adaptability to fit all requirements of nerve repair. As shown in Figure 1, the hydrogel can be made into a conduit and applied in peripheral nerve repair; the injectable hydrogel can match the requirement of brain nerve repair, and it can be directly used as an adhesive in spinal cord repair. As mentioned above, injectable hydrogel microspheres, as the new generation of the injectable hydrogel, are very promising applications in brain nerve repair as they have a good injectability and good designability, overcoming the defects of traditional injectable hydrogels, which require a precise control of the gelation time.

## 5. Modulation of Hydrogel Properties and Functions for Nerve Repair

In order to improve the repair effect of hydrogels in the process of nerve repair, improving the hydrogels to enhance their physical and chemical properties can largely promote axonal regeneration and nervous system reconstruction.

### 5.1. Morphology

The morphology of biomaterial substrates, such as pores, grooves, fibers and other surface morphological features, has a high impact on many aspects of cell function, including adhesion, proliferation, differentiation and migration [179,180]. As a kind of nerve tissue engineering material, hydrogels have a three-dimensional porous structure inside. The concentration and average pore size of hydrogels are regarded as important characteristic parameters that characterize the physical properties of the hydrogel, which are relevant to neurite extension in three dimensions. The pore size of the hydrogel is determined by its concentration, and the average porosity of the hydrogel exponentially decreases as the gel concentration increases [181]. George P. showed that there is a strong correlation between the ability of sensory ganglia to extend neurites and the hydrogel pore size. As the hydrogel concentration increases, the axon length decreases [182]. When hydrogel has a higher pore size, it can enhance the vitality, proliferation and migration of neural stem cells and promote its differentiation into neurons in the early stage of 3D culturing [183]. On the one hand, with the increase in average pore size, the hydrogel permeability increases, which is more conducive to the transfer and delivery of nutrients [184]. On the other hand, the specific surface area of hydrogels increased with the increase in average pore size [185]. Different types of cells have different corresponding range of pore sizes. There is a strong correlation between hydrogel-specific surface area and cell attachment. As the specific surface area of the hydrogel linearly decreases, the survival rate of attached cells decreases [186]. However, the decrease in hydrogel concentration may harm the mechanical properties and accelerate the degradation rate. Thus, the concentration and average pore size of hydrogels should be carefully optimized according to different requirements of various specific scenarios.

**Figure 8:**
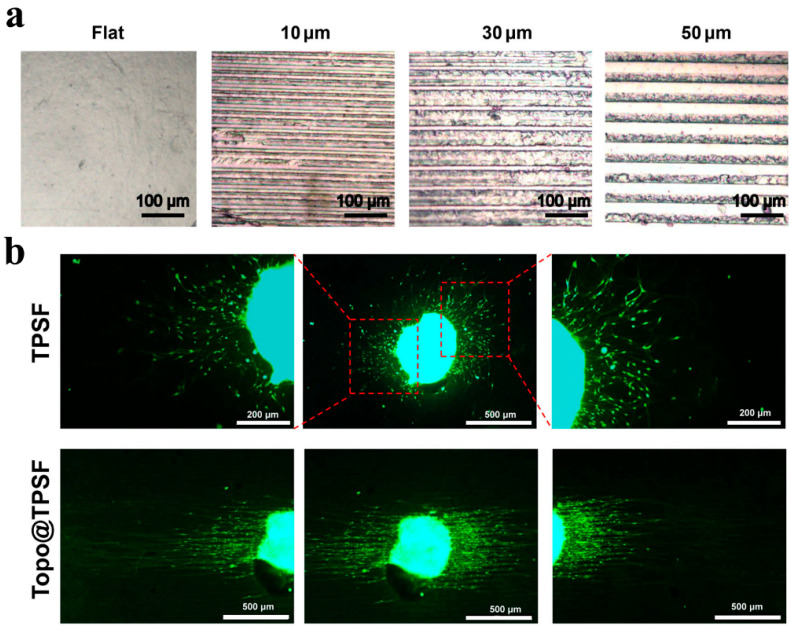
The hydrogels with a stable aligned micropattern have great potential for peripheral nerve regeneration [77]. (**a**) Microscopic images of microgrooves arranged on the surface of hydrogels; (**b**) fluorescence images of dorsal root ganglion cultured on TPSH (tough pure-silk fibroin hydrogels) and Topo@TPSF (micropatterned pure-silk fibroin hydrogels) after 3 days of incubation. (Adapted with permission from Ref. [77]. 2021, De Gruyter).

**Figure 9:**
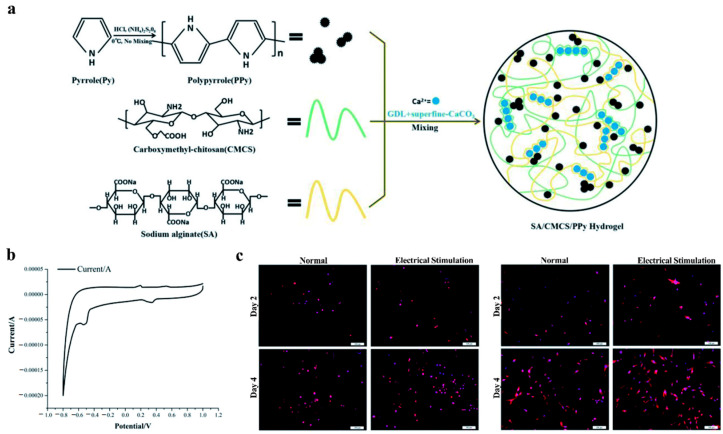
A conductive hydrogel doped with poly-pyrrole for peripheral nerve regeneration. (**a**) Preparation and structure of hydrogel; (**b**) cyclic voltammograms of the hydrogel; (**c**) representative fluorescence images RSC96 and BMMSC cells on hydrogels cultured for 2 and 4 days, and the electrical stimulation cell group stimulated with 100 mV for 2 h each 24 h. (Adapted with permission from Ref. [211]. 2018, Royal Society of Chemistry).

Changing the macroscopic shape or microscopic appearance of hydrogels is an important way to influence the increment and growth rate of nerve cells. In other words, it provides topological guidance for cells by simulating the microstructure of natural tissues [187]. The microscopic morphology of the hydrogel surface can be improved by simple preparation methods. As shown in Figure 8a, Gu used a PDMS stamp to prepare a pure-silk fibroin hydrogel with an aligned microgrooved topographic structure. This material can successfully regulate the growth of Schwann cells, promote axon growth, and guide neurite sprouting [77]. Reducing the spacing of microgrooves will increase the number of cells and nerve processes arranged along the groove direction [188,189]. The use of linear, multi-lumen guidance hydrogel conduits to repair peripheral nerve injury is a promising research direction. Conduits can promote axonal regeneration of the nervous system in a strikingly organized and linear fashion. Axons grow linearly through conduit channels [108]. On the contrary, axons are mostly radioactive in random and stochastic hydrogel conduits. As shown in Figure 9b, the hydrogel on the grooved surface showed the most effective ability to guide the neurite sprouting, and the neurite orientation angle was low. In contrast, neurons adhered to hydrogels without micropatterns extend neurite in all directions. The extension direction of neurites is random. In addition, the size of microchannels significantly contributed to the manipulation of neurite extension, Schwann cells migration, and fasciculation. Relatively smaller microchannels were even more beneficial for promoting directed neurite outgrowth and Schwann cells migration. Additionally, the large microchannels might provide sufficient space for neurites penetration and fasciculation, and the fiber bundle is thicker, which is beneficial for nerve maturation and functional recovery [190].

In the initial stage of nerve regeneration, the longitudinally oriented fibrin cables formed by fibrin can direct the migration and proliferation of Schwann cells and axonal regrowth. Experiments indicated that axons growing freely in a 3D hydrogel culture preferentially attach, turn and follow fibers with outgrowth rates and distances that far exceed outgrowth in a blank hydrogel [191]. By imitating natural fibrin cables, an instructive microenvironment can be created. Du prepared a three-dimensional hierarchically aligned fibrin nanofiber hydrogel, which displayed a hierarchically aligned topography. Rapid, directional cell adhesion and the migration of Schwann cells and dorsal root ganglion neurons were observed in vitro [192]. Many experimental results showed that the regeneration outcomes of the oriented fiber hydrogel in the process of nerve repair were superior to that of the random fibrin hydrogel [193]. By simulating the macroscopic or microscopic morphological characteristics in vivo, the biocompatibility of biomaterials can be improved. Providing additional physical guidance for the growth of cells and tissues can obtain better and faster treatments and repair effects.

### 5.2. Elastic Modulus

The elastic modulus of the hydrogel should meet the basic conditions of use. The elastic modulus depends on the network structure formed by the hydrogel. The network structure in turn controls the degradation behavior of the hydrogel, thereby controlling the release rate of the bioactive molecules loaded in the hydrogel [194]. If the elastic modulus of the hydrogel is low, it means that the three-dimensional network is sparse. It cannot fix the loaded cells, drugs and other substances inside, resulting in the problems, such as the inability to release bioactive molecules on demand and the loss of body fluids [105]. When used as matrix materials, the degradation rate and release rate of hydrogels should be compatible with the generation rate of cells and tissues.

On the other hand, the elastic modulus of the hydrogel and the tissue in the body to be repaired should be similar. Several studies described that the elastic modulus of the hydrogel microenvironment had a profound effect on cell structure and protein expression [195], affecting the behavior of a variety of cells, including stem cells, but these mechanical effects vary with different cell types [196]. The modulus of hydrogel influences the differentiation of mesenchymal stem cells into neurons, osteoblasts and myoblasts [197]. At the same time, it also affects the self-renewal and differentiation of neural stem cells [198]. The mechanical characteristics of human nervous system tissue are different. The elastic modulus of typical brain tissue ranges from 0.1 to 1 kPa [199]. The elastic modulus of the peripheral nerve is about 570 kPa [200]. The elastic modulus of spinal cord ranges from 1 to 1.4 MPa [201]. In fact, due to the complexity of the organism itself, the elastic modulus of different parts will be quite different under different testing and processing conditions, such as if the spinal cord has a dura mater during measurements, this will affect the results. In the literature, the same test was used to measure the elastic modulus of the material and the repaired part of the experimental object, so that the hydrogel can provide a specific range of elastic modulus, thereby obtaining a better repair effect. Taking the nerve repair of brain tissue as an example, neural stem cells can differentiate into three cell types: neurons, astrocytes and oligodendrocytes. Leipzig found that neuronal differentiation was favored on the softest surfaces, where the elastic modulus of the hydrogel is less than 1 kPa. Oligodendrocyte maturation and myelination was best when more mature neurons were present [202]. When the matrix elastic modulus is 500 Pa, neural stem cells optimally differentiate into neurons [203]. Mei discovered, after neural stem cells were encapsulated within hydrogels, that the rate of proliferation of neural stem cells decreased with the increase in the hydrogels modulus. The greatest enhancement in the expression of the neuronal marker b-tubulin III was found in soft hydrogels that had an elastic modulus comparable to that of brain tissues [199]. The more similar the mechanical properties of the hydrogel matrix and human tissues are, a specific differentiation of cells can be promoted, and a better repair effect can be achieved. The mechanical properties of hydrogels are characterized by various methods, such as compression resistance, swelling performance, loss modulus, etc. The elastic modulus mentioned in this paper is only one of the most concerned methods in the current research. In the process of actual research and future applications, we should pay more attention to the matching of material properties with the tissues to be repaired in all aspects.

### 5.3. Conductivity

The nervous system is one of the excitable tissues of the human body. In the nervous system, when neurons are stimulated to produce excitement, axons can transmit electrochemical impulses from neurons to their destinations, accompanied by the change in axon potential to realize the transmission of “information”. When the nervous system is damaged due to external forces or diseases, it will affect the control of the brain and spinal cord on other tissues, organs and muscles. Therefore, restoring electrical conduction is one of the most important goals of nerve repair [204,205].

At present, the most commonly used method to increase the electrical conductivity of hydrogels is to combine them with conductive polymers to form a conductive three-dimensional network. A large number of studies have shown that conductive materials have a good biocompatibility and have been widely used in biological tissue engineering [206,207]. There are two main methods to improve the electrical conductivity of hydrogels by using conductive polymers. One method is physical mixing, which adds conductive polymers to non-conductive hydrogels, such as graphene [208], polyaniline [209], polythiophene [210], poly-pyrrole [211], etc. The conductive materials are dispersed evenly in the form of monomers, fibers, lamellae or nanoparticles in hydrogels. The other is chemical modification, which directly synthesizes hydrogels with conductive polymers. Macromolecules are modified with conductive properties, such as grafting functional groups, to make the hydrogel itself have a higher conductivity through chemical cross-linking [200]. The addition of conductive materials can significantly promote the differentiation of neural stem cells into neurons; enhance the migration, proliferation and myelin formation of Schwann cells, including myelin specific gene expression and neurotrophic factor secretion [212]; promote the germination and growth of neural processes [213]; and improve the efficiency of nerve regeneration [214]. When using conductive biomaterials for tissue repair, “electrical stimulation” therapy is usually performed at the same time to obtain a better repair effect. Conducting hydrogels have shown the ability to stimulate and enhance neuron differential and outgrowth under electrical stimulation [215,216]. As shown in Figure 9, Bu prepared conductive sodium alginate and carboxymethyl chitosan polymer hydrogels. The hydrogels were cross-linked with calcium ions provided by the sustained release system consisting of D-glucose-d-lactone and superfine calcium carbonate, and the conductivity of the hydrogels was caused by doping with poly-pyrrole. The cyclic voltammograms confirmed that the hydrogels possessed an excellent electrochemical activity in Figure 9b. The advantages of conductive hydrogels in cell growth were verified by controlling the electrical stimulation of the cell experiments, as shown in Figure 9c. Reza synthesized a gelatin-graft-polyaniline/periodate-oxidized alginate hydrogel through the introduction of branched poly-ethylenimine. Compared with blank hydrogels, “electrical stimulation” significantly increased the expression of neural markers [217].

While using conductive polymers to improve the conductivity of hydrogel biomaterials, it is also necessary to pay attention to the influence of the amounts of conductive polymers on the physical properties and biosafety of the hydrogels. Gopal added amino-functionalized graphene into the hydrogel and found that the ordering and cross-linking of collagen molecules increased in the frozen hydrogels [218]. In the polyaniline hybrid hydrogel synthesized by Reza, the crosslinking density and storage modulus decreased with the increase in polyaniline content [217]. Many studies have shown that the physical properties of hydrogels, such as gel time, swelling rate, porosity and elastic modulus, are strongly related to the content of conductive polymers in gels [211]. This suggests to us that the enhancement of the conductivity of hydrogels should be balanced with their physical properties. In addition, many studies have shown that inorganic non-metallic nano materials such as graphene may have a dose-dependent biological toxicity to cells, tissues and the human body [219,220]. Although their mechanism of action is not clear, we should still pay attention to these potential risks. On the other hand, the preparation method of the conductive hydrogels should prevent the rejection reaction of the organism caused by the conductive material. If the composite material contains some toxic chemical residues, such as organic solvents or oxidants that cannot be removed during the preparation process, it will interfere with the material–cell interaction and eventually cause toxicity or cell death [221]. In addition, due to the long-term use of tissue engineering materials in the body, the changes in their properties will greatly reduce safety. Wang made polyaniline nanoparticles into a conductive coating through a layer-by-layer deposition to improve the conductivity of the nerve conduit. In the first four months, in vivo experiments in rats showed that, compared with the conduits without a polyaniline coating, the conduit with a polyaniline coating significantly improved the recovery of proximal compound muscle action potential in the regenerated nerve and was not inferior to the autograft group. In the later stage, when the nerve conduit matrix was degraded, the polyaniline coating ruptured, and fragments were formed in or around the regenerated nerve. The non-degradable polyaniline nanoparticles could induce cytotoxicity and reactive oxygen species and occupy the regeneration space of nerve tissue. When the polyaniline-coating was no longer intact, it stimulated inflammation [222]. At present, most animal experiments focus on the regeneration effect of nerves or the safety of materials in the short term. There is a lack of research on the long-term safety of conductive polymers.

Consequently, this shows that the modulation of physical properties can further enhance the nerve repair performance of the hydrogel carrier. Considering the strong designability of the hydrogel system, modulating the physical properties of the hydrogel becomes another powerful tool to improve the nerve repair performance of the hydrogel carrier in addition to various therapeutic agents. This also indicates that the modulation of physical properties must be considered when the new generation hydrogel carrier is designed and fabricated to repair nerves in the future.

## 6. Conclusions and Future Perspectives

In summary, hydrogel-based therapy has achieved significant progress in nerve repair. The excellent bio-safety of various hydrogels derived from both natural and synthetic sources confirmed that they can be used as carriers for nerve repair. Then, the wide loading capacity of therapeutic substances makes it possible to apply hydrogels in all nerve systems, including brain, spinal cord and peripheral nerves. Furthermore, it is also possible to load multiple drugs and modulate spatiotemporal sequential release in one carrier, which can definitely achieve a much more powerful therapeutic effect. Third, the shape variety of the hydrogel, resulting from various fabrication and processing techniques, can adapt to different scenarios of nerve repair. Injectable hydrogels can freely fill brain defects with any 3D shape and deliver a sufficient amount of therapeutic substances, such as neuroprotective agents, stem cells and various growth factors, to defects to maintain their long-term retention and play their roles in nerve repairing. Furthermore, Tregs is proven to be able to modulate local immune responses in brain defects so as to significantly promote on-site nerve regeneration. It is thought that Tregs therapy may be a very promising strategy for stroke rehabilitation. As mentioned above, the injectable hydrogel microspheres can promote cell growth and proliferation much better than tradition bulky injectable hydrogels. Thus, we are planning a series of experiments to be conducted in our lab in the near future in order to combine Tregs with an injectable hydrogel microsphere to repair brain nerve damage and loss after stroke, and we believe that Tregs therapy combined with injectable hydrogel microspheres may lead to successful stroke rehabilitation in the future. Finally, aside from the inert carrier, the physical properties of hydrogels, such as their moduli and conductivity, also play an important role in repairing nerves. Thus, modulating the physical properties is an effective and promising strategy to further enhance the nerve repairing ability of the hydrogel carrier, which might represent a major research direction in the future, producing some hydrogel carriers that can successfully repair the nerve and have the potential to be translated into a clinical setting.

Although some significant progress has been achieved in the repairing of the spinal cord and peripheral nerves, brain nerve repairing should be given more attention in the future. With the increase in the number of patients with brain diseases, such as stroke and Alzheimer’s disease, and the decline in the age of patients, our understanding and research of such diseases are also deepening. Through the exploration and integration of the existing findings, ways to improve quality of life and even the complete recovery of function for patients with such brain diseases is also an important research direction in the field of nerve repair. In addition, although hydrogel-based therapy in rodent models has produced promising results, further investigations on neuroplasticity, including neural networks and circuits, synaptic plasticity, axonal outgrowth, and dendritic spine density should be paid more attention in order to understand the nerve repairing process. Additionally, white matter damage and repair, as well as glia-mediated inflammatory responses, should be analyzed. Moreover, it is also very important that the cellular and molecular mechanisms underlying hydrogel-based therapy-driven neuroplasticity should also be comprehensively explored. All of these efforts may drive hydrogel-based therapy to cure stroke and other neurological diseases from lab to clinic in the future.
